# FGF2 alleviates LPS-induced acute lung injury by inhibiting ferritinophagy-mediated ferroptosis in AT2 cells via the Hippo-YAP signaling pathway

**DOI:** 10.3389/fimmu.2026.1788644

**Published:** 2026-04-17

**Authors:** Yan Wang, Pingjun Zhu, Yongkai Ding, Xinjie Han, Xi Wang, Sheng Wu, Siyuan Huai, Nan Li, Guogang Xu, Yingzhen Du

**Affiliations:** 1 Department of Emergency, Beijing Tsinghua Changgung Hospital, Tsinghua University, Beijing, China; 2 The Second Medical Center & National Clinical Research Center for Geriatric Diseases, Chinese People's Liberation Army (PLA) General Hospital, Beijing, China; 3 Chinese People's Liberation Army (PLA) General Hospital, Medical School of Chinese People's Liberation Army (PLA), Beijing, China; 4 Health Management Institute, The Second Medical Center, Chinese People's Liberation Army (PLA) General Hospital, Beijing, China; 5 Beijing Tsinghua Changgung Hospital, School of Clinical Medicine, Tsinghua University, Beijing, China; 6 Department of Radiotherapy, Oncology Division, Fifth Medical Center of Chinese People's Liberation Army (PLA) General Hospital, Beijing, China

**Keywords:** acute lung injury, ferroptosis, FGF2, Hippo-YAP signaling pathway, LPS

## Abstract

**Background:**

Ferroptosis of type II alveolar epithelial (AT2) cells plays a crucial role in the pathological progression of acute lung injury (ALI). Although fibroblast growth factor-2 (FGF2) has been shown to exert protective effects against ALI, the underlying mechanisms remain largely unexplored.

**Methods:**

The present study investigated the relationship between ferroptosis and FGF2 in the pathogenesis of ALI.

**Results:**

Our study found that FGF2 administration mitigated lung pathology, respiratory dysfunction, inflammation, and oxidative stress induced by lipopolysaccharide (LPS). Conversely, genetic knockout of FGF2 exacerbated lung injury, inflammation, oxidative stress, and ferroptosis. RNA sequencing and bioinformatics analyses identified ferroptosis as a key target of FGF2-mediated protection. Pharmacological induction of ferroptosis negated the protective effects of FGF2 on AT2 cells. Mechanistically, co-immunoprecipitation assays revealed that FGF2 suppressed ferritinophagy-associated changes by disrupting the interaction between NCOA4 and FTH1. Further investigation revealed that FGF2 modulated this interaction via the Hippo-YAP signaling pathway.

**Conclusion:**

Collectively, these results underscored the therapeutic potential of targeting the FGF2-mediated suppression of ferritinophagy-induced ferroptosis in treating LPS-induced ALI.

## Introduction

Sepsis, a potentially fatal condition characterized by an overwhelming host response to infection, often leads to multiple organ dysfunction syndromes, with the lungs being particularly vulnerable (1, 2). In the United States, approximately 200,000 patients annually suffer from acute lung injury (ALI), which is a common complication of sepsis, significantly contributing to the morbidity and mortality of individuals in intensive care units (3). ALI involves a complex pathophysiology, encompassing numerous cellular and molecular processes that ultimately result in severe lung dysfunction (4). Lipopolysaccharide (LPS), a component of the outer membrane of Gram-negative bacteria, plays a critical role in the development of ALI. It activates the Toll-like receptor 4 (TLR4) signaling pathway, triggering a robust immune response characterized by the release of pro-inflammatory cytokines and the recruitment of immune cells to the site of infection (5, 6). Despite advancements in supportive care, the development of effective treatment strategies remains hindered by the limited understanding of the underlying cellular and molecular mechanisms (7, 8). Given the incomplete understanding of the inflammatory processes involved in ALI, further research is essential to elucidate the precise molecular pathways responsible for LPS-induced ALI.

Type II alveolar epithelial (AT2) cells are vital for maintaining the integrity of the alveolar epithelium and facilitating lung repair following injury (9). In ALI, a severe respiratory condition characterized by widespread inflammation and disruption of the alveolar-capillary barrier, AT2 cells are essential for producing surfactant to maintain alveolar stability. They also serve as progenitor cells, differentiating into Type I alveolar epithelial cells to repair the damaged alveolar lining (10). The loss or dysfunction of AT2 cells exacerbates ALI by impairing the lung’s ability to repair damaged tissue, leading to more severe respiratory distress (2). Furthermore, AT2 cells interact with immune cells, such as macrophages, and modulate inflammatory responses by secreting anti-inflammatory molecules, including prostaglandin E2 and surfactant protein A (11). Given their pivotal role in both lung tissue regeneration and inflammation regulation, AT2 cells present a promising therapeutic target for improving outcomes in ALI.

As a form of programmed cell death distinct from apoptosis, ferroptosis is characterized by iron-dependent lipid peroxidation and the accumulation of reactive oxygen species (ROS). Recent research advances have significantly enhanced our understanding of its role in ALI. Increasing evidence suggests a crucial role of ferroptosis in the progression of ALI (12). Specifically, ferroptosis in AT2 cells has been shown to exacerbate the pathological changes associated with ALI. In LPS-induced ALI models, studies have demonstrated that AT2 cells exhibit increased markers of oxidative stress and cell death, such as elevated malondialdehyde (MDA) levels, accompanied by reduced levels of glutathione (GSH) and glutathione peroxidase 4 (GPX4) (13–15). During ALI, AT2 cells undergo changes related to ferroptosis. Interestingly, mechanisms exist to protect AT2 cells from oxidative damage in such conditions. For example, activation of the Nrf2/HO-1 pathway, known for its protective role against ferroptosis, has been shown to confer protective effects on AT2 cells in ALI (16). These findings underscore ferroptosis as a critical mechanism of AT2 cell injury in ALI, highlighting targeting ferroptosis as a promising therapeutic strategy for treating ALI.

The Fenton reaction, induced by iron overload, is critical for ferroptosis, leading to the generation of ROS (17). Elevated levels of free iron have been observed within the lung tissues of animals suffering from ALI (18). The Nuclear Receptor Coactivator 4 (NCOA4) mediates ferritinophagy, a process through which ferritin is degraded and its iron content is released (19). This released iron contributes to the labile iron pool and stimulates ferroptosis. Ferritin Heavy Chain 1 (FTH1), an essential component of the ferritin complex, binds selectively to the NCOA4 protein, which facilitates ferritinophagy. This interaction between NCOA4 and FTH1 plays a crucial role in maintaining intracellular iron homeostasis and may influence ferroptosis. However, how NCOA4-mediated ferritinophagy is related to LPS-induced ALI remains incompletely understood.

Fibroblast growth factor 2 (FGF2) is a multifunctional growth factor essential for various cellular processes, including cell proliferation, differentiation, and survival (20). In the context of ALI, FGF2 protects against lung damage and promotes tissue repair (21). Experimental studies have demonstrated that FGF2 signaling can alleviate lung injury, reduce inflammation, and enhance alveolar epithelial repair, underscoring its therapeutic potential in ALI. For example, FGF2 treatment could mitigate inflammation and alleviate oxidative stress in ALI models through the activation of the PI3K/Akt signaling pathway, which further decreases cell apoptosis and reduces inflammatory cytokine production (22). FGF2 has also been shown to modulate ferroptosis, particularly in the context of ischemia-reperfusion injury. It exerts protective effects by inhibiting oxidative damage and lipid peroxidation. In ischemia-reperfusion models, FGF2 has been demonstrated to reduce tissue injury and promote cell survival by activating antioxidant pathways such as the Nrf2/HO-1 axis, which subsequently inhibits ferroptosis (23).

The Hippo-YAP pathway plays a crucial role in sensing and regulating cellular environments and fate. In the nucleus, YAP and TAZ interact with the TEA domain (TEAD) family of transcription factors, specifically TEAD1-4, to modulate the expression of genes linked to cell growth and survival (24). YAP1 can effectively prevent ferritinophagy by disrupting the interaction between NCOA4 and FTH1, thereby reducing ferroptosis and alleviating ALI (19). FGF2 modulated the Hippo-YAP pathway in lung cells during sepsis, altering YAP phosphorylation status through mechanisms that may involve both canonical and non-canonical regulation (25). Our study explored whether exogenous FGF2 could inhibit ferritinophagy-induced ferroptosis through the Hippo-YAP pathway. The results indicated that FGF2 modulated YAP phosphorylation status, promoting YAP transcriptional activity, which in turn suppressed ferritinophagy and ferroptosis, thereby protecting against lung injury.

## Materials and methods

### Animals

Wild-type (WT) and FGF2-deficient (FGF2^-/-^) mice were obtained and bred as previously described (20). The study was approved by the Institutional Animal Care and Use Committee of the PLA General Hospital. Prior to the experiment, the mice were housed in a controlled environment with free access to food and water, allowing for a two-week acclimatization period. Mice were anesthetized with inhaled isoflurane using a precision vaporizer (induction: 3-4% isoflurane in oxygen/air at 0.8-1.0 L/min; maintenance: 1.5-2.5%). Adequate anesthesia was confirmed by loss of pedal withdrawal reflex and stable respiratory rate. ALI was induced by intratracheal administration of LPS (5 mg/kg, Sigma-Aldrich, USA) to 8-week-old male mice. Mice in the treatment group received 25 μg of recombinant growth factor protein (rFGF2) via intravenous injection 30 minutes before LPS administration according to previous study (20). The mice were injected with Verteporfin (100 mg/kg, i.p.; AdooQ Bioscience) 2 h and AZD4547 (2.5 mg/kg, i.p.; AbMole BioScience, Houston, TX, USA) before LPS injection (26, 27). Additionally, Fer-1 (5 mg/kg, HY100579, MedChemExpress) and Erastin (20 mg/kg, HY15763, MedChemExpress) were injected intraperitoneally daily for three consecutive days prior to the experiment. Twenty-four hours after LPS stimulation, the animals were euthanized for further analysis. Mice were euthanized by CO_2_ inhalation in a dedicated chamber with gradual fill (approximately 20-30% chamber volume per minute) until respiratory arrest, followed by a secondary physical method (cervical dislocation) to confirm death prior to tissue collection.

### Histological analysis

After the experiment, lung samples were collected and fixed in 4% PFA for 48 hours. Next, the samples were embedded in paraffin, sectioned, and stained with hematoxylin and eosin (H&E) to assess histopathological changes, following a previously established protocol.

### Cell counting in bronchoalveolar lavage fluid

Twenty-four hours after the LPS challenge, the mice were euthanized, and their BALF was collected. The BALF samples were centrifuged to isolate cellular components, and the resulting cell pellets were treated with Red Blood Cell Lysis Buffer for 5 minutes to remove erythrocytes. After two washes with ice-cold phosphate-buffered saline (PBS), the cells were collected and centrifuged again for 5 minutes at 4 °C. The isolated cells were then subjected to Wright-Giemsa staining for cytological analysis.

### Lung injury score

The degree of lung injury was evaluated based on four main aspects: thickening of the alveolar septum, presence of inflammation, hemorrhage, and edema. These parameters were assessed blindly using a semi-quantitative scale, with six random fields counted per slide (n = 6 per group). A 5-point scale was employed to evaluate alveolar septal thickening, hemorrhage, and edema: 0 for absent, 1 for mild, 2 for moderate, 3 for severe, and 4 for very severe. Inflammation was assessed by counting the total number of inflammatory cells per high-power field (HPF) with at least 100 HPFs analyzed per sample.

### Lung wet/dry ratios

Left lung samples were collected 24 hours following LPS stimulation. The samples were then perfused to remove any remaining blood, blotted dry, and immediately weighed to determine the wet weight. Subsequently, the samples were placed in a drying oven set at 80 °C for 48 hours to obtain the dry weight. The W/D ratio was calculated to assess tissue edema.

Arterial blood gas analysis.

PaO_2_ and PaCO_2_ in arterial blood were determined with the blood gas analyzer (ABL8000; Radiometer Copenhagen, Denmark) (3).

### Measurement of oxidative stress markers (ROS, MDA, SOD, GSH)

Twenty-four hours after LPS administration, the mice were sacrificed and their right lungs were excised. ROS contents were determined via dihydroethidium (Invitrogen, San Diego, CA, USA) staining and 2,7-dichlorodi-hydrofluorescein diacetate (Beyotime Institute of Biotechnology, Jiangsu, China). MDA content, SOD activity, and GSH level were evaluated with commercial kits (Beyotime Institute of Biotechnology, China).

### Cell culture and treatment

The primary AECII were separated from mice according to our previous study (3). In a humidified 5% CO_2_ incubator at 37°C, MLE-12 cells were cultured in DMEM/F12 medium supplemented with 10% fetal bovine serum, 1% penicillin, and 1% streptomycin. For LPS stimulation experiments, cells were treated with LPS (1 μg/mL) for 24 hours. Where indicated, rFGF2 (20 ng/mL) was added 30 minutes prior to LPS; Fer-1 (5 μM) and Erastin (10 μM) were co-administered with LPS; AZD4547 (10 μM) and Verteporfin (1 μM) were added 2 hours before LPS treatment.

### Western blot analysis

Lung tissue and cell samples were homogenized in ice-cold RIPA buffer (Solarbio, R0010) supplemented with protease inhibitor cocktail (Beyotime, P1048) and phosphatase inhibitor cocktail (Abcam, GR304037-28). Proteins were separated by 10% sodium dodecyl sulfate polyacrylamide gel electrophoresis and subsequently transferred onto nitrocellulose membranes. Next, the membranes were blocked with a 10% fat-free milk solution or bovine serum albumin (BSA, Beyotime, ST023) and incubated overnight at 4 °C with primary antibodies against FGF2 (1:1000, Abcam, ab222932), solute carrier family 7 member 11 (SLC7A11, 1:1000, Abcam, ab307601), GPX4 (1:1000, Abcam, ab125066), 4-hydroxynonenal (4-HNE, 1:1000, Abcam, ab48506), LC3II/I (1:1000, CST, 12741), NCOA4 (1:1000, CST, 53234), ferritin heavy chain 1 (FTH1, 1:1000, CST, 3998), LATS1 (1:1000, CST, 3477), MST1 (1:1000, CST, 3682), YAP (1:1000, CST, 14074), phosphorylated YAP (p-YAP, 1:1000, CST, 13008), and GAPDH (1:1000, CST, 2118). Following incubation, the membranes were incubated with HRP-conjugated IgG secondary antibody (Proteintech, SA00001-2) for 1 hour at room temperature. Band intensities were quantified using Bio-Rad Image Lab 3.0 software. For each target protein, the densitometric value was normalized to the corresponding GAPDH loading control band from the same membrane. For phosphorylated protein analysis, the ratio of phosphorylated protein to total protein (i.e., p-YAP/total YAP) was calculated from densitometric values obtained on the same membrane. For the LC3-II/LC3-I ratio, the densitometric values of LC3-II and LC3-I bands from the same membrane were used. All quantified data are presented relative to the control group, which was set to 1.0. All Western blot experiments were performed with n = 6 biologically independent samples per group.

### Measurement of ROS production *in vitro*

MLE-12 cells were incubated with the oxidation-sensitive fluorescent probe DCFH-DA (20 minutes, 37 °C). Next, the cells were washed with serum-free media to remove any excess probe. Cellular fluorescence was then measured using flow cytometry to assess ROS production.

### Immunofluorescence staining

MLE-12 cells were seeded on coverslips in well plates and incubated overnight. Following incubation, the cells were fixed for 15 minutes with 4% PFA. To block non-specific binding, the cells were treated with 5% bovine serum albumin (BSA) for 30 minutes at room temperature. The cells were then stored overnight at 4 °C and subsequently incubated with primary antibodies against Ferritin (1:500, Abcam, ab75973) and LAMP2 (1:500, Abcam, ab25631). After three washes with PBS (5 minutes each), the cells were incubated with fluorescence-conjugated secondary antibodies for one hour at room temperature. The coverslips were washed again with PBS to remove any excess secondary antibody and mounted on glass slides with 4’,6-diamidino-2-phenylindole (DAPI) reagent. Digital images were captured and analyzed using a Nikon microscope.

### Dihydroethidium staining

Frozen lung tissue sections (10 μm) were stained with 10 μM Dihydroethidium (DHE, Sigma-Aldrich, USA) to assess superoxide production. The sections were incubated in a humidified chamber at 37 °C for 30 minutes. Next, the tissue sections were washed with PBS and mounted with fluorescence mounting medium. Fluorescence microscopy was used to visualize DHE staining, with excitation at 488 nm and emission at 610 nm. The fluorescence intensity was quantified using image analysis software, and the average fluorescence intensity per field of view was calculated from at least five random fields per section.

### Dimension reduction, clustering and differentially expressed gene analysis

Publicly available Single-cell RNA sequencing data (GSE276682) from lung tissue under septic stress and control conditions in which ALI was induced by intraperitoneal injection of LPS were downloaded and re-analyzed in the current work for hypothesis-generating purposes as described in our previous study (28). Data quality was assessed by comparing key parameters, such as the number of molecules per cell (nCount RNA) and the number of detected genes per cell (nFeature RNA), with sequencing read counts. After filtering principal components, cell clustering was performed based on the unified manifold approximation and projection (UMAP) technique for dimensionality reduction, facilitating clear visualization of cell clusters. Statistically significant cell marker genes (adjusted p-values < 0.05) were identified and utilized to classify cell clusters. The cell marker genes from the DISCO database (https://www.immunesinglecell.org/) were compared with class-specific genes, enabling the identification of central genes and their distribution across various cell subpopulations.

### Real-time quantitative PCR

Total RNA was extracted from lung tissue or MLE-12 cells using the TRIpure Total RNA Extraction Reagent (ELK Biotechnology, EP013). cDNA synthesis was performed using the EntiLink™ 1st Strand cDNA Synthesis Kit (ELK Biotechnology, EP003). RT-qPCR was conducted using the EnTurbo™ SYBR Green PCR SuperMix (ELK Biotechnology, EP001). The expression levels of target genes were normalized to the housekeeping gene GAPDH.

### Fe^2+^ assay using ferroorange staining

MLE-12 cells were cultured overnight in 15 mm glass-bottom dishes for confocal microscopy visualization. The cells were then washed three times with Hank’s Balanced Salt Solution (HBSS). To assess Fe^2+^ levels, the cells were stained with 1 μmol/L Ferroorange (F374, DOJINDO, Japan) in HBSS for 30 minutes at 37 °C in a 5% CO_2_ atmosphere. The staining duration was carefully controlled for accuracy. Following staining, the cells were immediately imaged utilizing a Laser Scanning Confocal Microscope (FV3000).

### Statistical analysis

Data were analyzed using SPSS 25.0 (IBM) and are presented as means ± standard deviation (SD). All datasets were tested for normality using the Shapiro-Wilk test, and homogeneity of variance was verified by Levene’s test prior to ANOVA. For comparisons between two groups, a two-tailed unpaired Student’s t-test was used. For experiments involving multiple groups compared with a single control, one-way ANOVA followed by Dunnett’s multiple comparisons test was applied. For experiments involving pairwise comparisons among multiple groups, one-way ANOVA followed by Tukey’s multiple comparisons test was used. For comparisons involving two independent variables, two-way ANOVA followed by Tukey’s multiple comparisons test was employed. For Western blot densitometric data, the same statistical framework was applied to the normalized band intensity values. Significance was defined as P < 0.05.

## Results

### FGF2 expression was upregulated in lung tissue and AT2 cells during LPS-induced ALI

**Figure 1:**
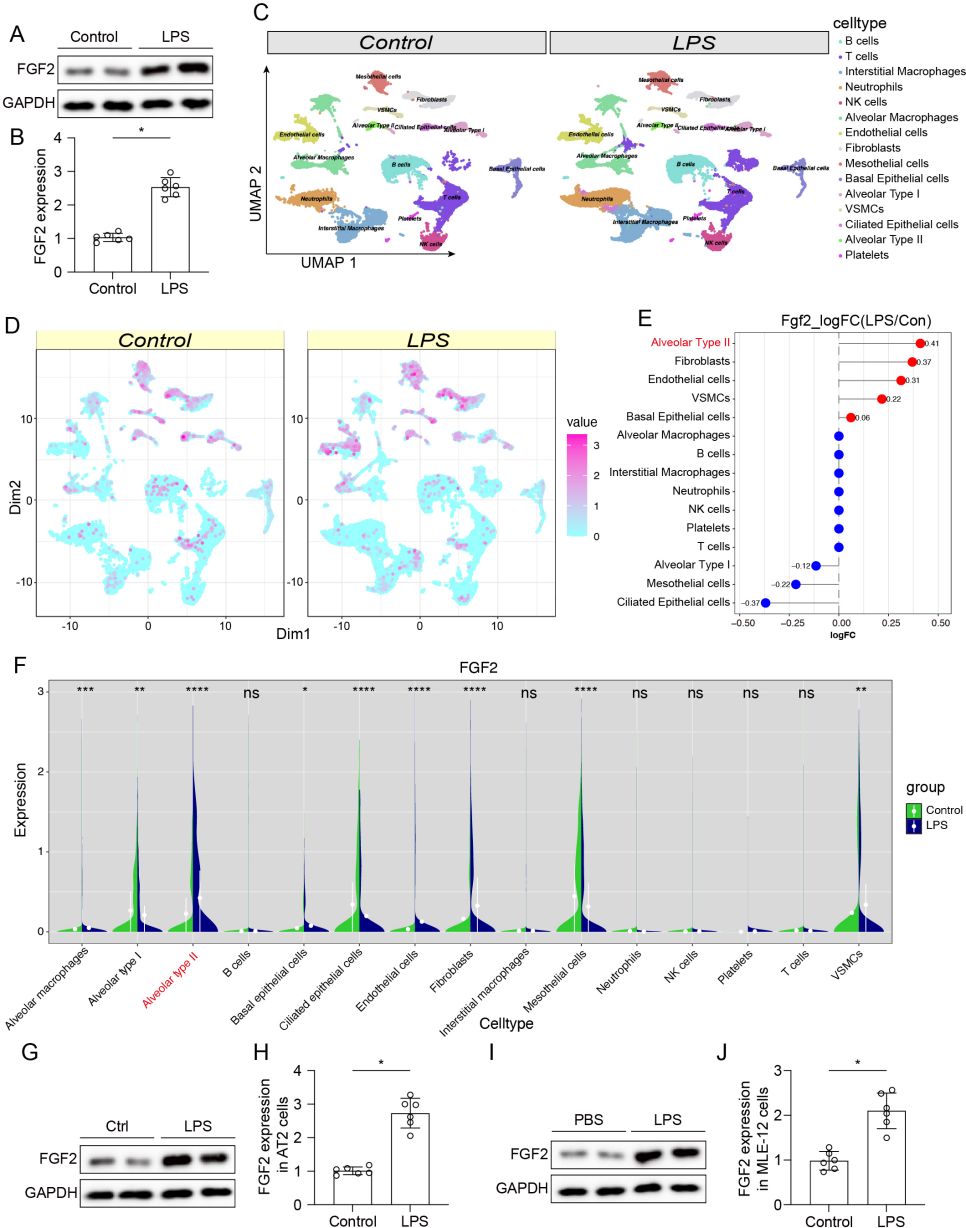
FGF2 expression was increased in lung tissue and AT2 cells during LPS-induced acute lung injury. **(A, B)** Representative Western blot **(A)** and quantification **(B)** of FGF2 protein levels in lung tissues from control (Ctrl) and LPS-treated mice. GAPDH served as the loading control. *P < 0.05. **(C)** UMAP plots depicting cell type clusters identified by single-cell RNA sequencing of lung tissues from control and LPS-treated mice. **(D)** UMAP plots showing FGF2 mRNA expression (color-coded by value) across various lung cell types from control and LPS-treated mice. **(E)** Dot plot showing the log2 fold change (LPS/Ctrl) of FGF2 mRNA expression in distinct lung cell populations. The dot size reflects the percentage of cells expressing FGF2, while the color represents the average expression level. **(F)** Violin plots displaying FGF2 mRNA expression levels in different lung cell types from control and LPS-treated mice. ****P < 0.0001. **(G, H)** Representative Western blot **(G)** and quantification **(H)** of FGF2 protein levels in AT2 cells isolated from control (Ctrl) and LPS-treated mice. GAPDH was used as the loading control. *P < 0.05. **(I, J)** Representative Western blot **(I)** and quantification **(J)** of FGF2 protein levels in MLE-12 cells treated with PBS (control) or LPS (1 μg/mL) for 24 hours *in vitro*. GAPDH served as a loading control. Data are presented as means ± SD (n = 6 biologically independent samples per group). Statistical analysis was performed using two-tailed unpaired Student’s t-test. *P < 0.05.

To investigate the role of FGF2 in LPS-induced ALI, its expression levels were assessed in a mouse model with LPS-induced ALI. Western blot analysis demonstrated that FGF2 protein expression was significantly upregulated in the lung tissues of mice challenged with LPS compared to controls (**Figures 1A, B**). To identify the specific cell types responsible for the upregulation of FGF2, existing single-cell RNA sequencing data from lung tissues of control and LPS-treated mice were reanalyzed. Unbiased clustering of the data revealed various cell populations, including B cells, T cells, NK cells, interstitial macrophages, neutrophils, alveolar macrophages, monocytes, endothelial cells, fibroblasts, basal epithelial cells, ciliated epithelial cells, alveolar type I cells, AT2 cells, and platelets (**Figure 1C**). Feature plots and dot plots showed a general increase in FGF2 mRNA expression across multiple lung cell types following LPS exposure (**Figures 1D, E**). However, violin plots revealed that FGF2 expression was significantly upregulated in AT2 cells from LPS-treated mice compared to controls (**Figure 1F**). To further confirm this finding at the protein level, western blot analysis of FGF2 was performed in isolated AT2 cells. The results confirmed a significant increase in FGF2 protein levels in the LPS treated group (**Figures 1G, H**). Similar findings were observed in MLE-12 cells (**Figures 1I, J**). These results suggest that FGF2 may play a critical role in the pathophysiology of ALI and warrant further investigation into its potential as a therapeutic target.

### Recombinant FGF2 administration ameliorated LPS-induced ALI

**Figure 2:**
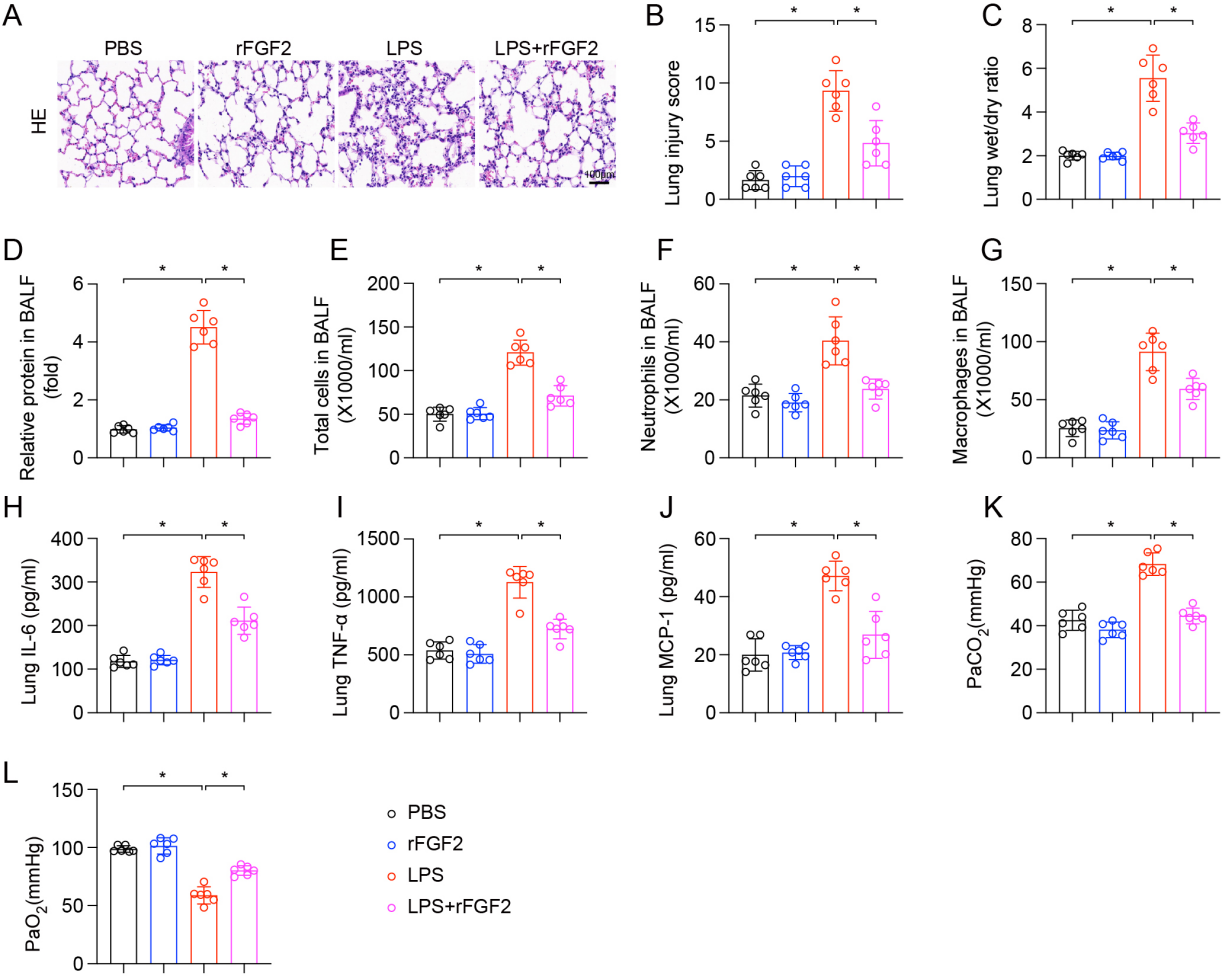
rFGF2 administration alleviated LPS-induced acute lung injury in mice. **(A)** Representative images of H&E stained lung sections from each group. **(B)** Quantification of lung injury scores. **(C)** Lung wet/dry weight ratio. **(D)** Relative protein concentration in BALF. **(E)** Total cell counts in BALF. **(F)** Neutrophil counts in BALF. **(G)** Macrophage counts in BALF. **(H-J)** Levels of IL-6 **(H)**, TNF-α **(I)**, and MCP-1 **(J)** in lung homogenates, as measured by ELISA. **(K, L)** Arterial partial pressure of PaCO_2_ **(K)** and PaO_2_ **(L)**. Data are presented as means ± SD (n = 6 biologically independent samples per group). Statistical analysis was performed using one-way ANOVA followed by Tukey’s multiple comparisons test. *P < 0.05.

To assess the protective role of FGF2 in LPS-induced ALI, recombinant FGF2 (rFGF2) was administered to mice prior to LPS challenge. Histopathological analysis of lung tissues via H&E staining revealed that LPS injection caused significant lung damage, characterized by thickening of the alveolar wall, oedema, and infiltration of inflammatory cells. However, pretreatment with rFGF2 significantly attenuated these pathological alterations (**Figure 2A**). In line with the histological findings, the LPS + rFGF2 group exhibited significantly lower lung injury scores compared to the LPS group (**Figure 2B**). Further analysis showed that rFGF2 treatment reduced LPS-induced pulmonary edema, as indicated by a significantly lower lung W/D weight ratio in the rFGF2-treated group (**Figure 2C**). Additionally, rFGF2 treatment decreased the increase in alveolar-capillary permeability, which was measured by total protein concentration in BALF (**Figure 2D**). LPS instillation led to a substantial influx of inflammatory cells into the alveolar space. Treatment with rFGF2 significantly reduced the total cell count in BALF (**Figure 2E**), as well as the numbers of neutrophils (**Figure 2F**) and macrophages (**Figure 2G**). In addition, the administration of rFGF2 reduced the inflammatory response in the lungs, as evidenced by significantly decreased levels of pro-inflammatory cytokines, including IL-6 (**Figure 2H**), TNF-α (**Figure 2I**), and MCP-1 (**Figure 2J**) in the lung homogenates. Finally, rFGF2 treatment improved arterial gas exchange in mice with LPS-induced ALI. LPS challenge resulted in a disruption of gas exchange, marked by an increase in PaCO_2_ and a decrease in PaO_2_. In contrast, rFGF2 pretreatment significantly reduced PaCO_2_ levels (**Figure 2K**) and increased PaO_2_ levels (**Figure 2L**) compared to the LPS-treated group. These results suggest that the administration of exogenous FGF2 effectively mitigates the severity of LPS-induced ALI by reducing lung injury, inflammation, and improving arterial gas exchange.

### FGF2 ameliorated LPS-induced ALI by suppressing ferroptosis

**Figure 3:**
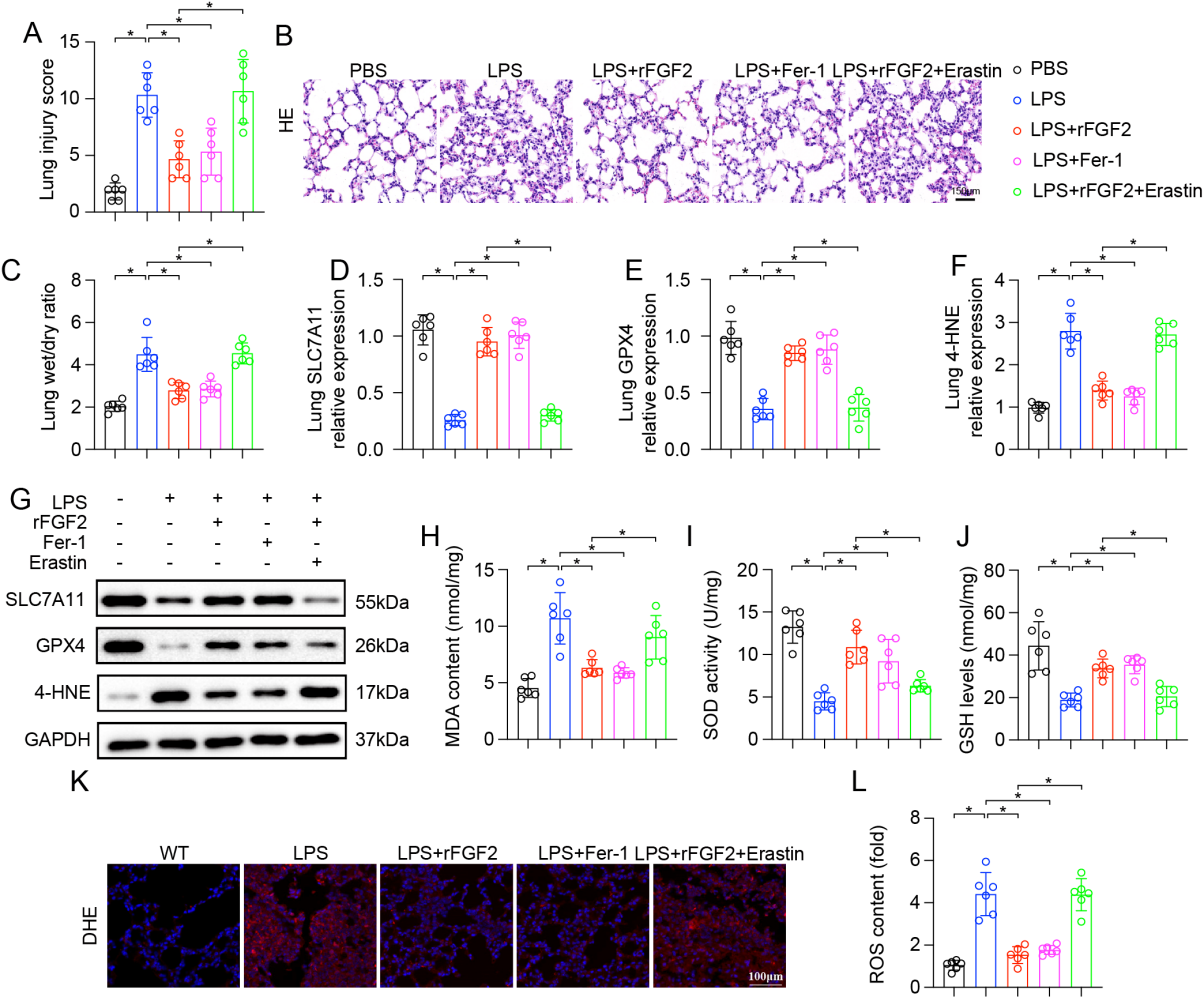
FGF2 protected against LPS-induced ALI by inhibiting ferroptosis (n=6 per group). **(A)** Quantification of lung injury scores. **(B)** Representative H&E-stained images of lung sections from each group. **(C)** Lung wet/dry weight ratio. **(D, E)** Relative mRNA expression of SLC7A11 **(D)** and GPX4 **(E)** in lung tissues, determined by RT-qPCR. **(F)** Relative mRNA expression of 4-HNE in lung tissues. **(G)** Representative Western blots showing protein levels of SLC7A11, GPX4, and 4-HNE in lung tissues. GAPDH was used as a loading control. **(H)** MDA content in lung tissues. **(I)** SOD activity in lung tissues. **(J)** GSH levels in lung tissues. **(K)** DHE staining for ROS in lung tissue sections. Scale bar = 100 µm. **(L)** Quantification of ROS content in lung tissues. Data are presented as means ± SD (n = 6 biologically independent samples per group). Statistical analysis was performed using one-way ANOVA followed by Tukey’s multiple comparisons test. *P < 0.05.

To explore whether the protective effect of FGF2 in LPS-induced ALI was mediated by the inhibition of ferroptosis, mice were administered with the ferroptosis inhibitor Fer-1 or the ferroptosis inducer Erastin, in combination with rFGF2 and LPS. Histological analysis and lung injury scoring revealed that, similar to rFGF2 treatment, Fer-1 significantly mitigated the lung damage induced by LPS. In contrast, the protective effects of rFGF2 were significantly reversed by the co-administration of Erastin, which exacerbated lung injury (**Figures 3A, B**). Moreover, rFGF2 and Fer-1 both reduced the LPS-induced increase in the lung W/D weight ratio, a marker of pulmonary edema, while Erastin counteracted the edema-reducing effects of rFGF2 (**Figure 3C**). Next, key markers of ferroptosis were examined. LPS treatment led to a significant downregulation of the core anti-ferroptotic proteins, SLC7A11 and GPX4, at both the mRNA and protein levels. This downregulation was reversed by treatment with either rFGF2 or Fer-1. However, the restorative effect of rFGF2 on SLC7A11 and GPX4 expression was abolished when Erastin was co-administered (**Figures 3D, E, G**). To further evaluate ferroptosis, we measured markers of lipid peroxidation, a key feature of ferroptosis. LPS treatment significantly increased the levels of 4-HNE, ROS, and MDA, while simultaneously decreasing the activity of the antioxidant enzyme superoxide dismutase (SOD) and the levels of GSH. Both rFGF2 and Fer-1 treatments effectively reversed these changes, indicating a reduction in oxidative stress and lipid peroxidation. The addition of Erastin abrogated the protective effects of rFGF2, leading to increased 4-HNE, ROS, and MDA levels, and decreased SOD and GSH levels (**Figures 3F–L**). DHE staining further confirmed that rFGF2 suppressed LPS-induced ROS production in lung tissue, an effect that was reversed by Erastin (**Figures 3K, L**). Collectively, these findings strongly suggest that FGF2-mediated protection in LPS-induced ALI is mediated, at least in part, through the suppression of ferroptosis.

### FGF2 suppressed LPS-induced ferroptosis, with AT2 cells identified as a primary target

**Figure 4:**
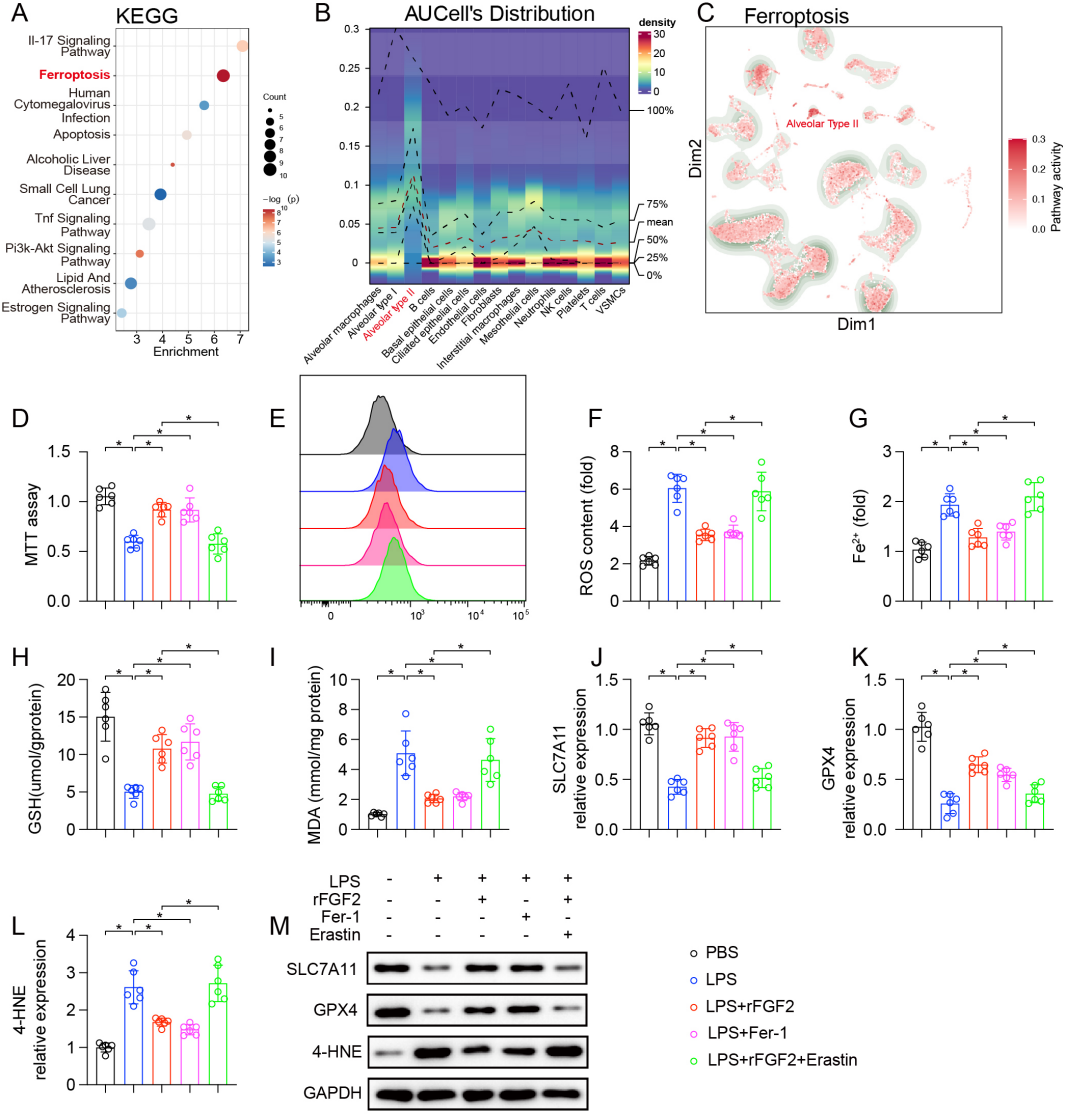
FGF2 protected AT2 from LPS-induced ferroptosis (n=6 per group). Analysis of scRNA-seq data from lung tissues. **(A)** KEGG pathway enrichment analysis of differentially expressed genes in AT2 cells from LPS-treated mice compared to controls. **(B)** AUCell distribution plot showing the activity score of the ferroptosis gene set across different lung cell types. **(C)** UMAP feature plot illustrating the activity of the ferroptosis pathway. Higher activity is indicated by red color. **(D–M)** *In-vitro* experiments on MLE-12 cells treated with PBS (control), LPS, LPS + rFGF2, LPS + Ferrostatin-1 (Fer-1), or LPS + rFGF2 + Erastin. **(D)** Cell viability assessed by MTT assay. **(E)** Representative flow cytometry histograms showing intracellular ROS levels measured by DCFHDA. **(F)** Quantification of ROS content. **(G)** Intracellular ferrous iron (Fe^2+^) levels. **(H)** GSH levels. **(I)** MDA content. **(J, K)** Relative mRNA expression of SLC7A11 **(J)** and GPX4 **(K)** in MLE-12 cells. **(L)** Relative mRNA expression of 4-HNE in MLE-12 cells. **(M)** Representative Western blots showing protein levels of SLC7A11, GPX4, and 4-HNE in MLE-12 cells. GAPDH served as a loading control. Data are presented as means ± SD (n = 6 biologically independent samples per group). Statistical analysis was performed using one-way ANOVA followed by Tukey’s multiple comparisons test. *P < 0.05.

To identify the cell type most affected by ferroptosis during LPS-induced ALI, single-cell RNA sequencing (scRNA-seq) data were examined. KEGG enrichment analysis of genes differentially expressed in lung tissue following LPS exposure identified ferroptosis as one of the most enriched pathways, alongside other inflammatory pathways (**Figure 4A**). We performed curve cell-level enrichment analysis (AUCell) scoring of the ferroptosis pathway for each single-cell subpopulation in the acute lung injury group and found that the AT2 subpopulation had the highest overall AUCell score among all subpopulations (**Figure 4B**). Furthermore, a feature plot visually confirmed that the highest activity of the ferroptosis pathway was localized to the AT2 cells cluster (**Figure 4C**). These findings strongly suggest that AT2 cells are a primary site of ferroptosis during ALI.

To validate whether FGF2 directly protects AT2 cells by inhibiting ferroptosis, *in-vitro* experiments were performed using MLE-12 cells. An MTT assay demonstrated that LPS treatment reduced MLE-12 cell viability, which was restored by treatment with either rFGF2 or the ferroptosis inhibitor Fer-1. The protective effect of rFGF2 was abrogated by co-treatment with the ferroptosis inducer Erastin (**Figure 4D**). Key markers of ferroptosis were then assessed. LPS treatment resulted in a significant increase in ROS (**Figures 4E, F**), intracellular ferrous iron (Fe^2+^) (**Figure 4G**), and the lipid peroxidation marker MDA (**Figure 4I**), along with a depletion of the antioxidant GSH (**Figure 4H**). These effects were significantly reversed by treatment with either rFGF2 or Fer-1. However, the addition of Erastin counteracted rFGF2’s protective effects, which further confirmed the involvement of ferroptosis. At the molecular level, LPS treatment downregulated the expression of the anti-ferroptotic proteins SLC7A11 and GPX4, while increasing the expression of the lipid peroxidation product 4-HNE. Treatment with either rFGF2 or Fer-1 rescued SLC7A11 and GPX4 expressions, and reduced 4-HNE levels. Again, the protective effects of rFGF2 were abolished by co-treatment with Erastin (**Figures 4J–M**). In summary, these findings demonstrated that AT2 cells are likely a major population undergoing ferroptosis in LPS-induced ALI based on scRNA-seq and *in vitro* evidence, and that FGF2 suppresses the ferroptosis program in AT2-like cells in culture.

### FGF2 inhibited LPS-induced ferritinophagy in AT2 cells

**Figure 5:**
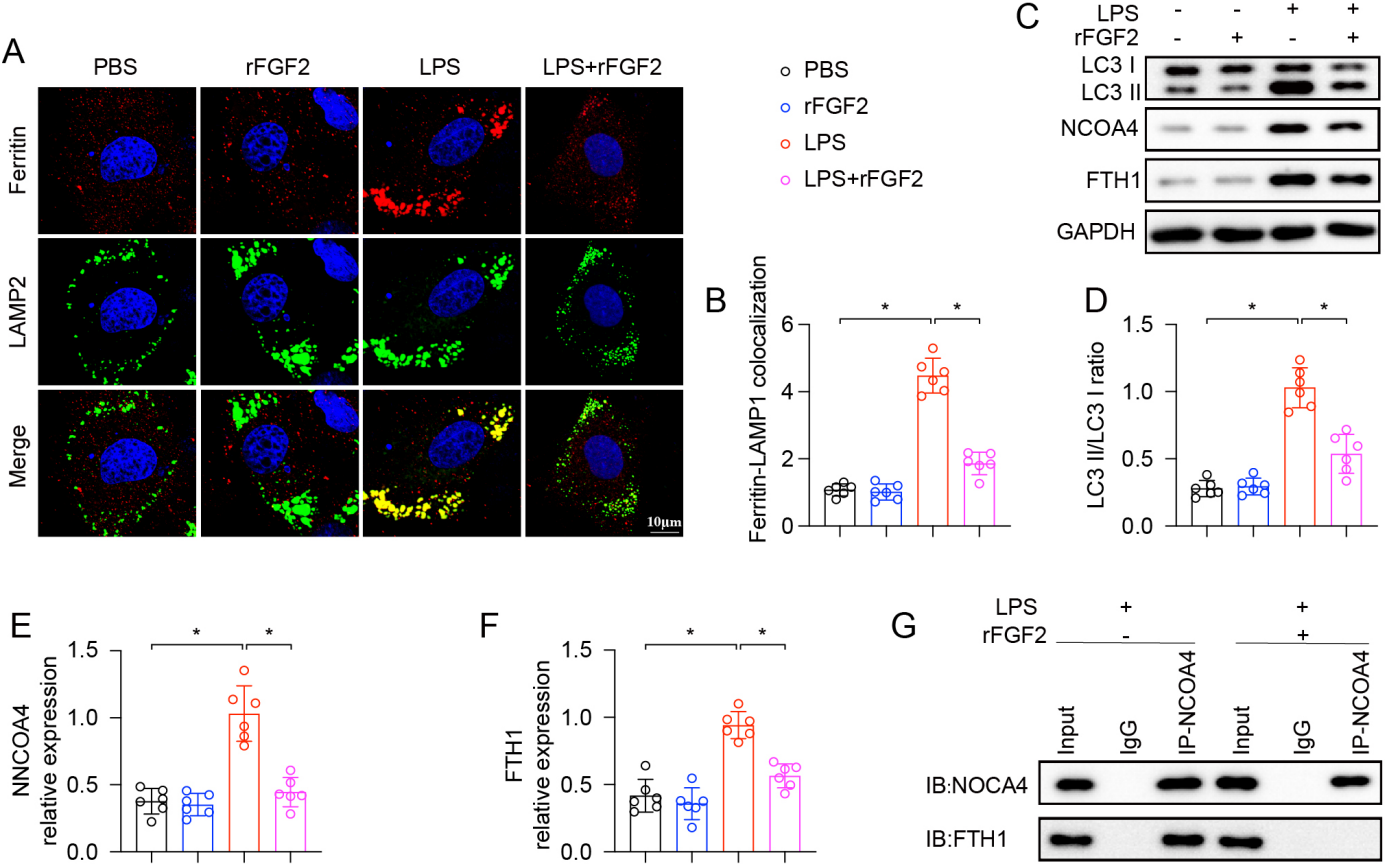
FGF2 suppressed ferritinophagy in AT2 cells treated with LPS (n=6 per group). **(A)** Representative immunofluorescence images of MLE-12 cells stained for ferritin (red), LAMP2 (lysosomal marker, green), and nuclei (DAPI, blue). Yellow in the merged images indicates colocalization. Scale bar = 10 µm. **(B)** Quantification of the colocalization between ferritin and LAMP2 from the images in **(A)**. **(C–F)** Representative Western blots for LC3-II, LC3-I, NCOA4, and FTH1 in MLE-12 cells. GAPDH was used as a loading control. **(G)** Co-IP of NCOA4 and FTH1. Data are presented as means ± SD (n = 6 biologically independent samples per group). Statistical analysis was performed using one-way ANOVA followed by Tukey’s multiple comparisons test. *P < 0.05.

Given the close association between ferroptosis and iron metabolism, the potential role of FGF2 in protecting against ALI by regulating ferritin autophagy was explored. Immunofluorescence staining was performed among MLE-12 cells to assess the overlap between ferritin and lysosomes, marked by LAMP2. In LPS-treated cells, there was an obvious increase in the colocalization of ferritin with LAMP2, suggesting the transport of ferritin to lysosomes for degradation. Treatment with rFGF2 markedly inhibited this process (**Figures 5A, B**). To further elucidate the underlying mechanism, Western blot analysis was carried out to examine key proteins involved in ferritinophagy. LPS treatment was associated with changes consistent with enhanced ferritinophagy, including an increased LC3-II/LC3-I ratio, upregulation of NCOA4 and a corresponding reduction in FTH1 levels. Treatment with rFGF2 significantly attenuated the LPS-induced elevation in the LC3-II/LC3-I ratio and NCOA4 expression, while preventing the degradation of FTH1 (**Figures 5C–F**). To investigate whether rFGF2 affects the interaction between NCOA4 and ferritin, co-immunoprecipitation assays were performed. In LPS-treated MLE-12 cells, NCOA4 strongly interacted with FTH1. However, in cells co-treated with both LPS and rFGF2, the interaction between NCOA4 and FTH1 was substantially reduced (**Figure 5G**). These results collectively suggest that FGF2 may alleviate LPS-induced AT2 cell injury, at least in part, by suppressing NCOA4-mediated ferritinophagic processes and preserving FTH1 levels.

### FGF2 inhibited ferritinophagy through modulation of the Hippo-YAP signaling pathway

**Figure 6:**
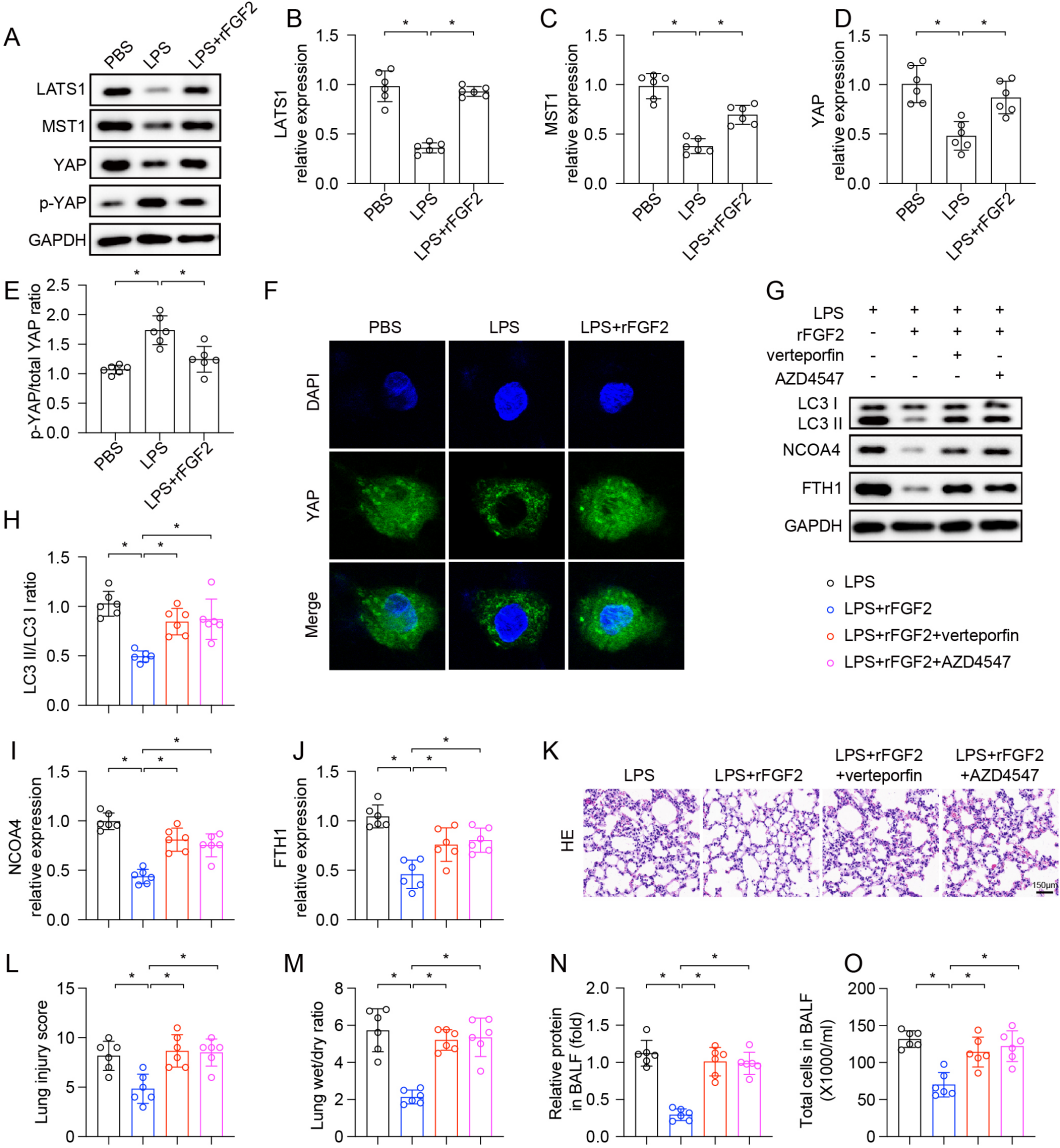
The protective effect of FGF2 against ferritinophagy was mediated by the Hippo-YAP signaling pathway (n=6 per group). **(A–E)** Representative Western blots for LATS1, MST1, total YAP, and p-YAP to total YAP protein levels. **(F)** Immunofluorescence staining showed YAP expression and its localization. **(G–J)** Representative Western blots for LC3-II, LC3-I, NCOA4, and FTH1. **(K)** Representative H&E stained images of lung sections. Scale bar = 100 μm. **(L)** Quantification of lung injury scores. **(M)** Lung wet/dry weight ratio. **(N)** Relative protein concentration in BALF. **(O)** Total cell counts in BALF. Data are presented as means ± SD (n = 6 biologically independent samples per group). Statistical analysis was performed using one-way ANOVA followed by Tukey’s multiple comparisons test. *P < 0.05.

Recent studies have implicated the Hippo-YAP pathway as a potential link between FGF2 and NCOA4-mediated ferritinophagy (25, 29, 30). To uncover the mechanism linking FGF2 to ferritinophagy, we investigated the Hippo-YAP pathway. LPS treatment reduced the total protein levels of MST1 and LATS1. Notably, despite the reduction in total Hippo kinase levels, the p-YAP/total YAP ratio was significantly increased, indicating enhanced YAP phosphorylation and functional inactivation through a mechanism that may involve non-canonical regulation or compensatory kinase activity (**Figures 6A–E**). Treatment with rFGF2 restored MST1 and LATS1 protein levels and reduced the p-YAP/total YAP ratio, suggesting promotion of YAP nuclear translocation and transcriptional activation. Given the importance of YAP’s nuclear localization for its function, we examined its role in YAP localization through immunofluorescence staining. We discovered FGF2 not only elevated YAP expression in LPS-damaged cells but also facilitated its nuclear translocation (**Figure 6F**).

To determine whether YAP inactivation contributed to the inhibitory effect of FGF2 on ferritinophagy, *in-vitro* experiments were performed on MLE-12 cells using verteporfin, a pharmacological inhibitor of YAP, and AZD4547, an inhibitor of the FGF receptor (FGFR). As previously demonstrated, rFGF2 treatment suppressed the LPS-induced increase in the LC3-II/LC3-I ratio and NCOA4 expression, while preserving FTH1 levels. Notably, direct inhibition of YAP with verteporfin reversed the rFGF2-mediated suppression of ferritinophagy markers, restoring LC3-II/LC3-I ratio and NCOA4 levels toward those observed with LPS treatment alone. Conversely, blocking the FGF receptor with AZD4547 completely abrogated the ability of rFGF2 to inhibit ferritinophagy, returning the levels of LC3, NCOA4, and FTH1 to those seen with LPS treatment alone (**Figures 6G–J**). To validate these findings *in vivo*, we examined the effects of pathway inhibitors on rFGF2-mediated protection. Co-administration of the YAP inhibitor verteporfin with rFGF2 abrogated the protective effects of rFGF2, as demonstrated by worsened histological features and increased lung injury scores (**Figures 6K, L**). Verteporfin co-treatment also exacerbated pulmonary edema (**Figure 6M**), capillary leakage (**Figure 6N**), and inflammatory cell infiltration (**Figure 6O**). Similarly, the FGFR inhibitor AZD4547 significantly blocked the protective effects of rFGF2 in LPS-induced lung injury. These results collectively demonstrate that FGF2 signals through its receptor to modulate YAP phosphorylation status and promote YAP nuclear activity, thereby inhibiting ferritinophagy.

### FGF2 knockout exacerbated LPS-induced ALI and ferroptosis

**Figure 7:**
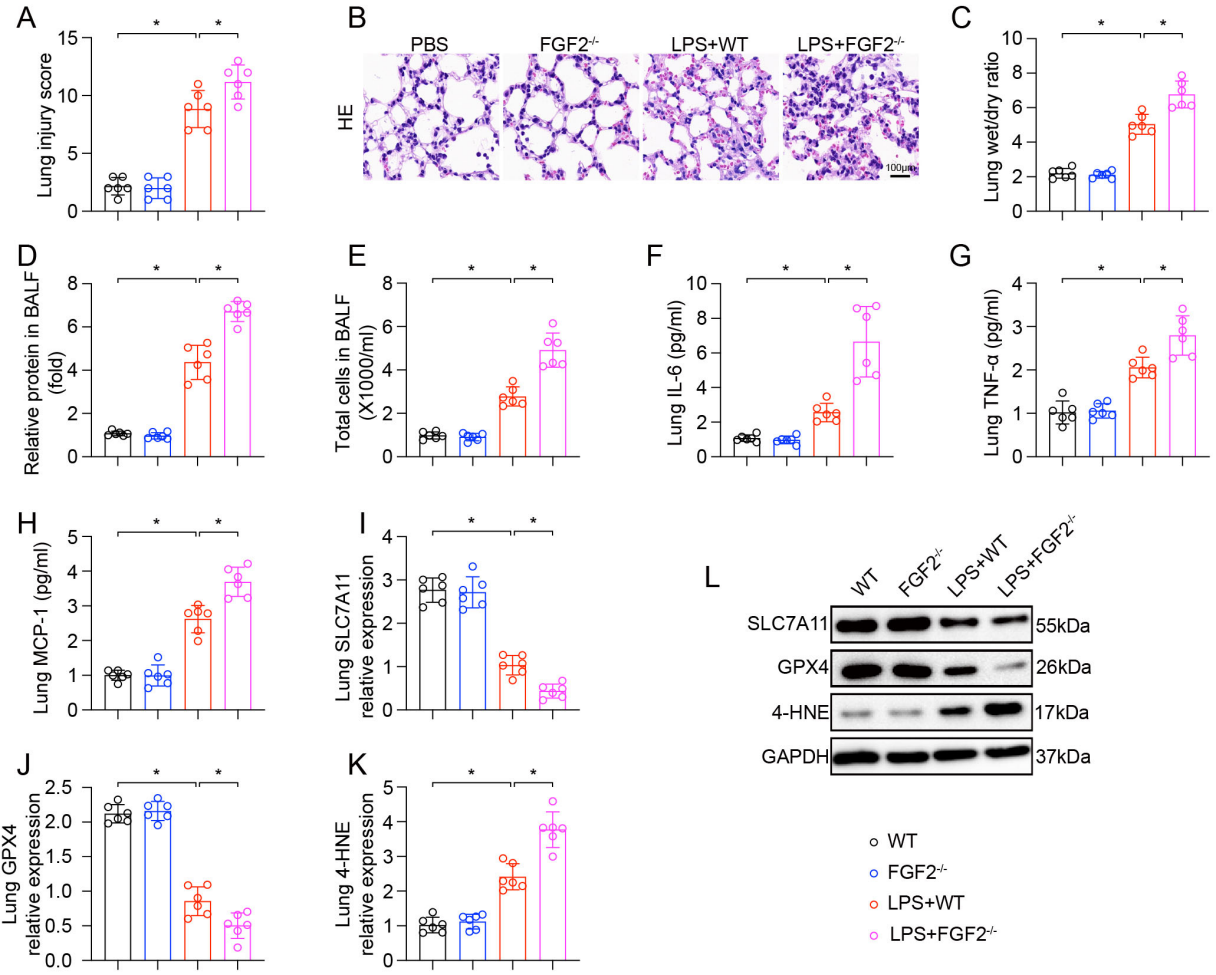
Genetic deletion of FGF2 worsened LPS-induced acute lung injury and ferroptosis (n=6 per group). **(A)** Quantification of lung injury scores. **(B)** Representative H&E-stained images of lung sections from each group. **(C)** Lung wet/dry weight ratio. **(D)** Relative protein concentration in BALF. **(E)** Total cell counts in BALF. **(F–H)** Levels of IL-6 **(F)**, TNF-α **(G)**, and MCP-1 **(H)** in lung homogenates. **(I–L)** Relative protein expression of SLC7A11, GPX4, and 4-HNE in lung tissues, quantified from Western blots. GAPDH served as a loading control. Data are presented as means ± SD (n = 6 biologically independent samples per group). Statistical analysis was performed using two-way ANOVA followed by Tukey’s test. *P < 0.05.

To confirm the protective role of endogenous FGF2 within ALI, FGF2^-/-^ mice were utilized. Both WT and FGF2^-/-^ mice were challenged with LPS. While FGF2^-/-^ mice exhibited no abnormalities at baseline, they showed a significantly exacerbated response to LPS challenge compared to WT mice. Histological analysis revealed more severe lung damage in the LPS-treated FGF2^-/-^ mice, which was reflected in a markedly higher lung injury score (**Figures 7A, B**). Furthermore, FGF2^-/-^ mice displayed greater pulmonary edema, as evidenced by an increased lung W/D ratio (**Figure 7C**). The absence of FGF2 also resulted in more pronounced alveolar-capillary barrier disruption, as indicated by a higher protein concentration in the BALF (**Figure 7D**). In line with the increased tissue damage, the inflammatory response was more severe in FGF2^-/-^ mice. These mice showed a greater influx of total inflammatory cells into the BALF (**Figure 7E**) and significantly higher IL-6, TNF-α, and MCP-1 levels in their lung tissue compared to LPS-treated WT mice (**Figures 7F–H**). Finally, we assessed markers of ferroptosis. Following LPS challenge, the lungs of FGF2^-/-^ mice exhibited a more pronounced downregulation of the anti-ferroptotic proteins SLC7A11 and GPX4, as well as a significantly higher level of the lipid peroxidation marker 4-HNE, compared to WT mice (**Figures 7I–L**). These findings provide strong genetic evidence that endogenous FGF2 exerts a crucial effect on LPS-induced ALI, primarily by mitigating the inflammatory response and suppressing ferroptosis.

## Discussion

This study investigated the role of FGF2 in the regulation of ferroptosis within the context of ALI. The key findings were as follows: (1) FGF2 expression was upregulated in lung tissue following LPS stimulation, with scRNA-seq analysis identifying AT2 cells as a prominent source. (2) rFGF2 treatment improved LPS-induced ALI and suppressed ferroptosis markers both *in vivo* and in AT2-like cells *in vitro*. (3) In contrast, genetic deletion of FGF2 exacerbated both lung injury and ferroptosis. (4) *In vitro* evidence suggests that FGF2 inhibits ferroptosis in AT2-like cells by suppressing ferritinophagy. (5) Mechanistically, FGF2 was associated with reduced YAP phosphorylation and enhanced YAP nuclear accumulation, which correlated with disruption of the NCOA4–FTH1 interaction and suppression of ferritinophagy. Ferroptosis has been implicated in the pathogenesis of ALI, contributing to alveolar epithelial cell death, and previous studies have shown that inhibiting ferroptosis can reduce lung damage and inflammation in ALI models (12). Our findings are consistent with these observations and further demonstrate that FGF2-associated suppression of ferritinophagic markers is linked to reduced ferroptosis. To our knowledge, this study is the first to establish a direct link between FGF2 and the regulation of ferritinophagy in ALI, underscoring the potential therapeutic role of FGF2 in mitigating ferroptosis and alleviating ALI.

A notable observation in our study is that endogenous FGF2 was significantly upregulated in lung tissue and AT2 cells following LPS challenge, yet these mice still exhibited severe lung injury. This apparent paradox can be explained by interpreting the upregulation of FGF2 as a compensatory, endogenous stress response that is quantitatively insufficient to counteract the overwhelming inflammatory and oxidative injury cascade. This phenomenon is well-documented for other cytoprotective molecules such as HO-1 and Nrf2, which are commonly upregulated during ALI but fail to fully prevent tissue damage at endogenous levels (16). In the context of FGF2, Wang et al. demonstrated that FGF2 is significantly elevated during influenza A virus-induced ALI as part of the host defense response, yet endogenous levels alone are insufficient to prevent lung damage (20). Our data from FGF2^−/−^ mice directly support this interpretation: the absence of the compensatory FGF2 response led to significantly worse lung injury, inflammation, and ferroptosis compared to wild-type littermates (**Figure 7**). Therefore, the administration of supraphysiological doses of exogenous rFGF2 amplifies this inherent protective response beyond the threshold required to effectively suppress ferritinophagy and ferroptosis.

FGF2 plays a multifaceted role in lung injury and inflammation. It is essential for epithelial repair and maintenance of lung epithelial integrity following injury (21), exhibits immunomodulatory properties in chronic inflammatory airway diseases (31), and reduces pulmonary fibrosis by inhibiting collagen deposition and epithelial-mesenchymal transition (32). In sepsis-induced ALI, FGF2 alleviates inflammation, reduces capillary leakage and macrophage infiltration, and enhances survival (33) while FGF2 deletion from macrophages exacerbates sepsis-induced ALI by promoting the release of pro-inflammatory cytokines (34). Our study demonstrated a consistent upregulation of FGF2 expression within the lungs of mice with LPS-induced ALI, with FGF2 predominantly expressed in AT2 cells, aligning with prior studies and suggesting its involvement in regulating alveolar epithelial function (35, 36). We also observed a broader distribution of FGF2-positive cells beyond AT2 cells, potentially including immune cells that are known to play significant roles in ALI progression. Future studies utilizing AT2-specific knockdown models will be crucial for elucidating the cell-autonomous role of FGF2 within AT2 cells.

Recent evidence suggests that FGF2 is crucial for the modulation of ferroptosis. FGF2-mediated protection against ferroptosis are primarily mediated through its ability to mitigate oxidative stress and enhance cellular antioxidant defenses. Specifically, FGF2 activates the Nrf2/HO-1 signaling pathway, which counteracts ferroptosis by reducing ROS and lipid peroxidation (23). Additionally, FGF2 has been shown to preserve mitochondrial integrity under stress conditions, thereby reducing mitochondrial-derived ROS, which are key contributors to ferroptosis (37, 38). Our study further demonstrated that FGF2 suppressed ferritinophagy in LPS-induced ALI, consistent with its role in limiting iron-dependent damage. The multifaceted actions of FGF2 suggest that it not only inhibits the drivers of ferroptosis but also enhances cellular resilience against oxidative and iron-dependent damage, positioning FGF2 as a promising protective target for diseases associated with ferroptosis.

NCOA4 is a selective cargo receptor that mediates the autophagic degradation of FTH1 within the autophagosome, facilitating the release of free iron into the cytosol (39). This process is tightly regulated, as excessive ferritinophagy can expand the labile iron pool, exacerbating oxidative stress and ultimately promoting ferroptosis. Our findings suggest that FGF2 exerts its anti-ferroptotic effects, at least in part, by modulating the NCOA4–FTH1 axis, directly disrupting their interaction as demonstrated by co-immunoprecipitation assays. While the convergence of five independent lines of evidence—including the LC3-II/LC3-I ratio, NCOA4 expression, FTH1 degradation, ferritin–LAMP2 colocalization, and NCOA4–FTH1 co-immunoprecipitation—strongly supports the conclusion that FGF2 modulates selective ferritinophagy, we acknowledge that formal autophagic flux assays were not performed. Future studies should incorporate bafilomycin A1 or chloroquine treatment to block lysosomal degradation, which would enable definitive discrimination between increased autophagosome formation and impaired autophagic flux. The LC3-II/LC3-I data should therefore be interpreted with this methodological caveat in mind. The identification of ferritinophagy as a specific target of FGF2 expands our understanding of its protective mechanisms, providing a foundation for developing targeted interventions in conditions where ferroptosis plays a central role. While ferritinophagy has been studied in the contexts of cancer and neurodegeneration, its role in LPS-induced ALI and the specific inhibitory effect of FGF2 on this process highlight a novel aspect of the molecular mechanisms underlying ALI.

The key mechanistic observation of our study is that FGF2 blocks ferritinophagy by acting along the Hippo-YAP signaling pathway. We emphasize that our data demonstrate a functional association between FGF2 treatment and altered YAP phosphorylation status, rather than definitive activation of the canonical MST1/LATS1 kinase cascade. The term ‘Hippo-YAP modulation’ is used throughout to reflect this distinction. The observed decrease in p-YAP/total YAP ratio upon rFGF2 treatment, combined with immunofluorescence evidence for YAP nuclear translocation (**Figure 6F**), indicates that FGF2 functionally promotes YAP transcriptional activity; however, the upstream signaling events connecting FGFR to YAP dephosphorylation remain to be fully elucidated and may involve PI3K-AKT-mediated inhibition of Hippo kinases, altered phosphatase activity, or non-canonical mechanisms. This modified interpretation that FGF2 promotes YAP nuclear activity to suppress ferritinophagy completely aligns with the analysis of Zhang et al., who showed that sepsis-induced ALI was countered due to YAP1 activity which down-regulates NCOA4–FTH1 interaction and inhibits ferritinophagy-mediated ferroptosis (19). Similarly, Qi et al. reported that in non-alcoholic fatty liver disease it is YAP that inhibits ferritinophagy by NCOA4 (30) and Sun et al. described the YAP/TAZ–ferritinophagy axis in placental ferroptosis (29). Taken together, these results create a comprehensive model in which active nuclear YAP is an inhibitory regulator of ferritinophagy in various pathological situations and in which FGF2 serves as an upstream positive regulator of YAP nuclear activity. A key limitation of this study is the absence of genetic manipulation of NCOA4. Although our co-immunoprecipitation data demonstrate that FGF2 disrupts the NCOA4–FTH1 interaction, and our pharmacological experiments (verteporfin and AZD4547) support the proposed signaling axis, NCOA4 knockdown or knockout experiments were not performed. Such genetic approaches—for instance, siRNA-mediated NCOA4 silencing in MLE-12 cells followed by assessment of whether FGF2’s anti-ferroptotic effects are diminished—would provide definitive causal evidence for the NCOA4-dependent mechanism. Without these experiments, the possibility that FGF2 suppresses ferroptosis through additional NCOA4-independent mechanisms cannot be excluded. Future studies should incorporate NCOA4 loss-of-function models as a priority.

Our verteporfin experiments provide direct functional evidence that YAP–TEAD transcriptional activity is essential for FGF2’s anti-ferritinophagy effects. Verteporfin specifically blocks the YAP–TEAD protein–protein interaction, the principal transcriptional output of the Hippo pathway (30). The complete reversal of FGF2-mediated NCOA4 suppression and FTH1 preservation by verteporfin (**Figures 6F–I**) demonstrates that these effects require YAP-dependent gene transcription. However, we acknowledge that the specific downstream transcriptional targets of the YAP–TEAD complex responsible for disrupting the NCOA4–FTH1 interaction remain to be identified. Future studies should measure canonical YAP–TEAD target genes, such as CTGF (CCN2) and CYR61 (CCN1), and perform transcriptomic analysis following FGF2 treatment to delineate the specific transcriptional program through which YAP suppresses ferritinophagy.

Regarding the intermediate signaling events connecting FGFR to the Hippo kinase cascade, several plausible mechanisms can be proposed based on the existing literature. FGFR activation has also been shown to upregulate the RAS-MAPK and PI3K-AKT cascades that control Hippo kinases. In particular, AKT has been shown to directly phosphorylate MST1 at inhibitory sites to inhibit the Hippo kinase cascade and promote activation of the YAP pathway. Furthermore, FGFR signaling also modulates Hippo pathway activity via cytoskeletal rearrangement and Rho GTPase-dependent regulation of LATS1/2 kinase activity. In ovarian granulosa cells, FGF2 downregulates Hippo signaling, activating YAP and allowing cell growth (25). Additionally, bidirectional mechanisms of interaction between FGF2 and YAP have been shown in cellular settings, where YAP promotes FGF2 responsiveness by upregulating FGFR expression in neural stem cells (40) and promotes FGF2 transcription and secretion in gliomas in response to radiation-induced YAP activation (41). This highlights the complex relationship between FGF2 and YAP signaling that goes beyond the one-way dynamic model described in this work. The precise intermediate signaling between FGFR and MST1/LATS1 in AT2 cells in the case of ALI needs to be studied further in future investigations.

Notably, co-administration of Erastin nearly completely abolished the protective effects of rFGF2, restoring ferroptosis markers to levels comparable to those observed with LPS treatment alone rather than producing an intermediate phenotype. This outcome can be rationalized by considering the distinct nodes at which FGF2 and Erastin act within the ferroptosis regulatory network. FGF2 operates primarily upstream by suppressing ferritinophagy, thereby limiting the expansion of the labile iron pool that catalyzes lipid peroxidation. Erastin, by contrast, acts at a convergent yet mechanistically independent node: it directly inhibits the system Xc^-^ transporter (SLC7A11), depleting intracellular cysteine and GSH, and consequently inactivating GPX4-dependent lipid peroxide detoxification. Because Erastin disables the terminal antioxidant checkpoint, residual intracellular iron derived from ferritinophagy-independent sources, such as transferrin receptor-mediated uptake, remains sufficient to sustain lipid peroxidation even when ferritinophagy is partially attenuated by FGF2. This interpretation is further supported by the pharmacological doses of Erastin employed in this study (20 mg/kg *in vivo*; 10 μM *in vitro*), which were selected to achieve maximal SLC7A11 inhibition. Collectively, the ability of Erastin to override *FGF2-mediated protection* reinforces the conclusion that the primary anti-ferroptotic mechanism of FGF2 in this model is ferritinophagy inhibition rather than direct augmentation of the GPX4-dependent antioxidant defense.

At the therapeutic dose of 25 μg administered in this study (single pre-treatment dose), our macroscopic and histological evidence of toxic effects such as increased tissue size, fibrotic changes, and atypical growth was missing during 24 hours. Thus, FGF2 demonstrated robust FGF2-mediated anti-ferroptosis defense activity in this study. For an acute condition such as ALI/ARDS, short-term administration of FGF2 forms the clinically relevant scenario here, greatly alleviating theoretical risks associated with prolonged exposure to a growth factor. Importantly, Guzy et al. showed that FGF2 is necessary for epithelial repair, yet does not induce lung fibrosis following bleomycin-induced damage, which gives reassurance as to the less likely association of short-term FGF2 treatment with fibrogenic mechanisms (21). In addition, extensive toxicity studies including longer study lengths, dose-response analysis, and possible off-target effects (as well as angiogenic and mitogenic hazards) should be sought prior to any clinical translation of FGF2-based therapies to ALI.

The current study has several important limitations that should be acknowledged. First, rFGF2 was administered before LPS challenge, representing a preventive rather than therapeutic approach. Post-treatment studies administering rFGF2 at various time points after injury onset are needed to evaluate its translational potential. Second, the *in vivo* ALI model utilized intratracheal LPS, which produces direct lung injury rather than the indirect lung injury seen in systemic sepsis. While intratracheal LPS is an established model endorsed by the American Thoracic Society, it may not fully recapitulate the pathophysiology of sepsis-associated ALI. Additionally, the scRNA-seq dataset used for exploratory analysis was derived from an intraperitoneal LPS model, introducing a methodological inconsistency with our primary *in vivo* model, although the key findings were independently validated in the intratracheal model. Third, the arterial blood gas analyses (PaCO_2_ and PaO_2_) reflect gas exchange rather than formal pulmonary function, and the elevated PaCO_2_ values may reflect species-specific ventilatory responses in anesthetized mice rather than clinical ALI/ARDS patterns. Fourth, formal autophagic flux assays using lysosomal inhibitors (e.g., bafilomycin A1 or chloroquine) were not performed. While multiple convergent lines of evidence support our ferritinophagy interpretation, the LC3-II/LC3-I data alone cannot definitively distinguish between increased autophagosome formation and impaired autophagic degradation. Fifth, NCOA4 genetic manipulation (knockdown or knockout) was not performed, and thus the causal dependence of FGF2’s anti-ferroptotic effects on the NCOA4-mediated pathway remains to be definitively established. Sixth, while we measured total LATS1 and MST1 protein levels, we did not assess their phosphorylated (active) forms; therefore, the upstream kinase dynamics driving the observed changes in YAP phosphorylation status remain to be resolved. Seventh, Hippo-YAP signaling and ferritinophagy were primarily characterized in MLE-12 cells *in vitro*. Whole-lung Western blots cannot resolve cell-type-specific signaling. Definitive demonstration of the FGF2–Hippo-YAP–ferritinophagy axis within AT2 cells *in vivo* requires AT2-specific conditional genetic models (e.g., Sftpc-CreERT2; YAPfl/fl) or single-cell-resolution proteomic approaches. Lastly, Hippo-YAP signaling was not examined in FGF2^-^/^-^ mouse lung tissue, which would have provided important genetic corroborative evidence for the proposed pathway.

## Conclusions

The study demonstrated that FGF2 significantly alleviated LPS-induced lung injury and suppressed ferroptosis, with scRNA-seq and *in vitro* data suggesting that AT2 cells are a major cellular target of this protective mechanism. Our data suggest that FGF2 suppresses ferritinophagy-associated changes by disrupting the NCOA4–FTH1 interaction, thereby attenuating ferroptosis. These findings, while supported by multiple convergent lines of evidence, remain to be confirmed by formal autophagic flux assays and NCOA4 genetic loss-of-function studies. These findings highlight FGF2 as a promising protective target warranting further investigation for LPS-induced ALI, suggesting its potential as a novel treatment strategy for this condition.

## Data Availability

The raw data supporting the conclusions of this article will be made available by the authors, without undue reservation.
